# Varicella zoster virus outbreak in a long-term care unit of a tertiary care hospital in northern India

**DOI:** 10.1017/S0950268824000712

**Published:** 2024-05-13

**Authors:** Rushika Saksena, Bonnie J. Thomas, Ruma Das, Sunita Nagpal, Prem R. Suri, Ranjan K. Wadhwa, Aashish Choudhary, Rajni Gaind, Ekta Gupta

**Affiliations:** 1 Department of Microbiology, VMMC and Safdarjung Hospital, New Delhi, India; 2 Department of Microbiology, AIIMS, New Delhi, India; 3 Department of Physical Medicine and Rehabilitation, VMMC and Safdarjung Hospital, New Delhi, India; 4 Department of Virology, ILBS, New Delhi, India

**Keywords:** Varicella zoster, Chickenpox, Hospital-acquired (nosocomial) infections, Outbreaks

## Abstract

Nosocomial outbreak of varicella zoster virus (VZV) has been reported when susceptible individuals encounter a case of chicken pox or shingles. A suspected VZV outbreak was investigated in a 50-bedded in-patient facility of Physical Medicine and Rehabilitation in a tertiary care multispecialty hospital. A 30-year-old female patient admitted with Pott’s spine was clinically diagnosed with chicken pox on 31 December 2022. The following week, four more cases were identified in the same ward. All cases were diagnosed as laboratory-confirmed varicella zoster infection by PCR. Primary case was a housekeeping staff who was clinically diagnosed with chicken pox 3 weeks prior (9 December 2022). He returned to work on eighth day of infection (17 December 2022) after apparent clinical recovery but before the lesions had crusted over. Thirty-one HCWs were identified as contacts a and three had no evidence of immunity. Two of these susceptible HCWs had onset of chickenpox shortly after first dose of VZV vaccination was inoculated. All cases recovered after treatment with no reported complications. VZV infection is highly contagious in healthcare settings with susceptible populations. Prompt identification of cases and implementation of infection prevention and control measures like patient isolation and vaccination are essential for the containment of outbreaks.

## Introduction

Varicella zoster virus (VZV) causes a highly communicable, usually self-limiting endemic infection in children commonly called chickenpox. But the infection can have a more serious clinical course in adults, immunocompromised, sick and debilitated long-term care patients [1]. The virus can spread quickly when people co-exist in proximity, like schools, colleges, long-term care facilities, hospital wards, and ICUs. When patients with already weak immune system due to multiple pathologies contract the disease, treating them can be especially challenging [2].

Nosocomial outbreak of VZV have been reported when susceptible individuals encounter a case of chicken pox or shingles [2]. Susceptible individuals are patients and healthcare workers (HCWs) with no evidence of immunity to varicella. Evidence of immunity to varicella is defined by the Advisory Committee on Immunization Practices (ACIP) as fulfilment of any of the following four criteria: (a) documentation of two doses of varicella vaccine, (b) laboratory evidence of immunity or laboratory confirmation of disease, (c) diagnosis or verification of a history of varicella disease by a healthcare provider, and (d) diagnosis or verification of a history of herpes zoster by a healthcare provider [3]. If the number of individuals without immunity against VZV is high, in that setting disease can spread rapidly with a secondary attack rate of 80% in community and 90% in hospital setting [4]. According to Minhas et al., there were about 269 chickenpox outbreaks accounting for 27257 cases documented between January 2015 and May 2021 in India [5]. Prevention of outbreaks can be achieved by increasing the population of immunized people in such settings and by implementing appropriate infection prevention and control measures [6, 7]. We report an outbreak of VZV at Physical Medicine and Rehabilitation (PMR) ward of Vardaman Mahavir Medical College and Safdarjung Hospital that started on 31 December 2022 and ended on 27 February 2023. The effective identification and control of the outbreak by the hospital infection control team curtailed the spread of infection. The objective of this outbreak report is to describe the measures implemented in controlling the spread of the infection by our hospital infection control team.

## Setting

Vardhman Mahavir Medical College and Safdarjung Hospital is a 2800 bedded multispecialty tertiary care hospital in Delhi, India. Although the hospital is primarily an acute care facility, the Department of PMR admits patients requiring long-term care and has an in-patient facility with 50 beds distributed in 7 cubicles. The ward has an open layout with Nurse: Patient ratio of 1:5 and Doctor: Patient ratio of 1:3. Average bed occupancy was about 60%. There are in total of 30 HCWs in the ward including 8 doctors, 13 nurses, 4 nursing orderlies, and 5 housekeeping staff. At the time of the outbreak, 17 patients were admitted for various rehabilitative care.

The study was approved by the institutional ethics committee (IEC/VMMC/SJH/Project/2022-11/CC-316) and hospital administration.

## Outbreak report

A 30-year-old female patient diagnosed with Pott’s spine with paraplegia admitted on 6 December 2022 presented with complaints of fever and a pustular rash all over the body on 31 December 2022. A clinical diagnosis of VZV infection was made after dermatology opinion and the patient was immediately isolated. This was identified as the index case. On 2 January 2023, three more patients complained of similar symptoms and were diagnosed with VZV clinically. All patients were cohorted in an isolation ward with adequate ventilation and sunlight as no negative pressure room was available in the facility. Treatment with acyclovir was also initiated.

The hospital infection control (HIC) team was informed of the suspected outbreak on 2 January 2023 and the team visited the PMR ward. Vesicular fluid and scab material samples were collected in viral transport medium from all four patients. The vesicular lesions were unroofed and the base of the lesion was vigorously swabbed using a flocked nylon swab to collect epithelial cells and vesicular fluid. Samples were sent to Virology Lab, Department of Microbiology at AIIMS, New Delhi for detection of VZV DNA by PCR. Swabs are processed immediately once received in the virology laboratory. They were rotated several times against the VTM tube walls to ensure maximal transfer of material into the fluid, and the swabs were discarded. The tubes were then centrifuged for 5 min at 3000 rpm. DNA was extracted using the QIAmp DNA extraction kit (Qiagen, Hilden, Germany) following manufacturer’s instructions. The processed samples were stored in −80 °C freezer until testing was performed. VZV DNA was detected by real-time PCR using the Fast Track Diagnostics (Siemens Healthineers, Fast Track Diagnostics Luxembourg) kit. This kit is a multiplex PCR kit which simultaneously detects herpes simplex virus 1 (HSV-1), herpes simplex virus 2 (HSV-2), and VZV. All samples tested positive for VZV DNA and an outbreak of nosocomial varicella zoster was confirmed.

HIC team consisting of clinical microbiologists and infection control nurses (ICNs) instructed the HCWs regarding contact and airborne precautions to be taken during care for these patients. These included strict compliance to hand hygiene, PPE including gloves, gown and N95 mask, dedicated patient care equipment and a separate washroom for the isolated cohort. Only one caregiver was allowed to stay with each patient after determining a history of past infection with chickenpox. New admissions to the ward were restricted and any patient requiring admission was placed in a separate in-patient facility.

HIC conducted extensive interviews of the staff in the ward to identify the probable source. Detailed history was taken from nurses and doctors who were involved with direct patient care as well as from patient attendants. Other staff like nursing orderlies, housekeeping staff and security guards not involved in direct patient care were also asked about any recent history of fever and rash. We found that one of the housekeeping staff was diagnosed clinically with VZV infection on 9 December 2022. He returned to work on the eighth day of infection (17 December) after apparent clinical improvement with no episodes of fever and malaise but before the lesions had crusted over. He was identified as the likely primary case.

On 5 January, one more patient showed symptoms of VZV, and samples were sent for PCR which came positive. To identify HCWs who were immune to varicella zoster, serum samples were collected from all HCWs for VZV IgG titers. History of chickenpox and VZV immunization was also elicited. Eighteen out of 31 HCWs gave a history of chickenpox and only one had taken VZV vaccine. Three HCWs had low VZV titers and were identified as persons with no evidence of immunity. These individuals were provided with the first dose of VZV vaccine on 8 January, 0.5 ml s.c injection of live attenuated vaccine of Oka strain (VARIVAX, Merck, Rahway, NJ). They were also reassigned to care for unexposed patients and asked not to interact with positive patients and their caregivers. Only immune HCWs were assigned to take care of infected patients. All HCWs were asked to self-monitor for any symptoms of chickenpox and report immediately to HIC if infection is suspected.

**Figure 1.**
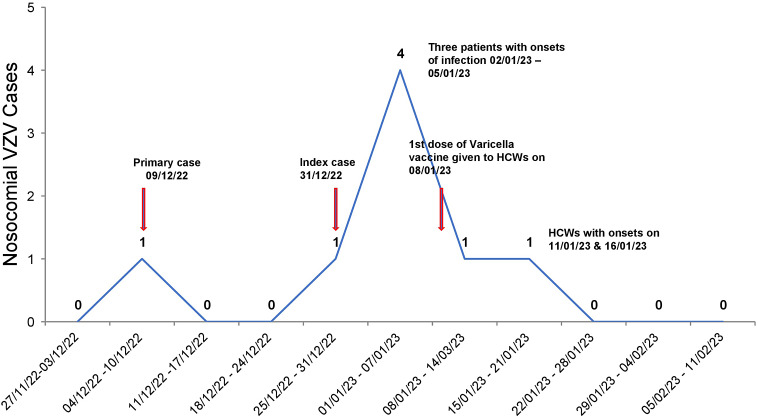
The epidemiological curve of VZV cases in Physical Medicine and Rehabilitation ward.

**Figure 2.**
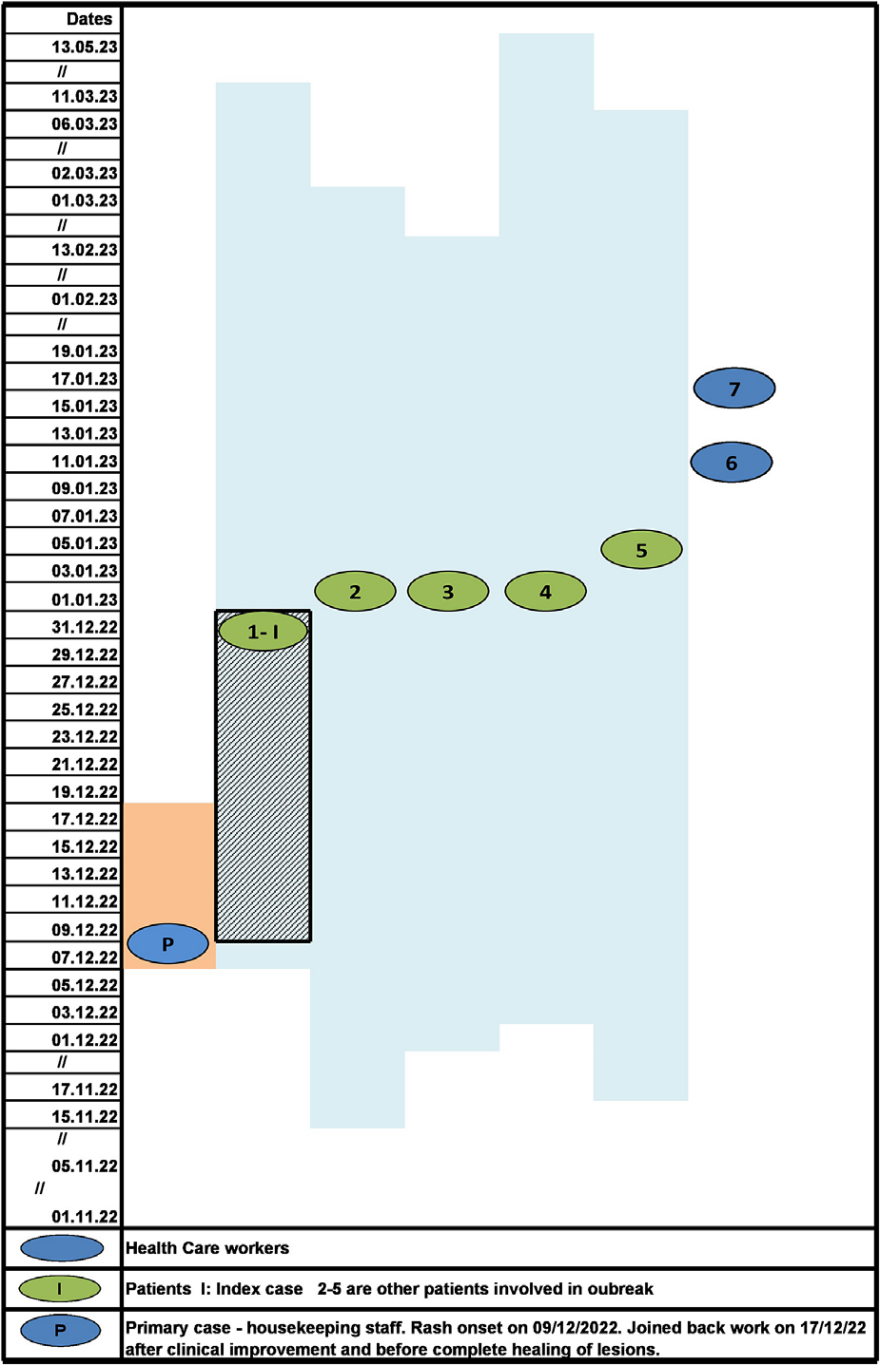
Evolution of VZV outbreak in Physical Medicine and Rehabilitation ward with probable mode of transmission. *Note:* Blue shaded area: duration of hospital stay of each patient is indicated by date of admission and discharge. Blue shaded area with grid: maximum incubation period of Index case. Orange shaded area: infectious period of primary case (housekeeping staff). Initially, the probable mode of transmission was airborne as there was no direct contact of primary case with patients and further transmission between patients and HCW was both airborne and contact during patient care. The reported incubation period is 10–21 days. The likely date of exposure of index cases to primary case, accordingly, should lie between 10 December 2022 and 31 December 2022 depicted as grid lines. Therefore, index case was likely infected after the HCW joined back work following clinical response but before lesions had completely healed (orange shaded area depicts when HCW was on leave)

Despite early implementation of IPC practices and initiation of vaccination, two of the nursing staff with no evidence of immunity against VZV developed the disease on 11 and 16 January 2023. The HCWs were placed on medical leave to self-quarantine, till the lesions were completely crusted. All patients recovered from VZV infection and were eventually discharged. Infected HCWs had only mild disease and joined back work after 21 days when lesions had healed and were no longer infectious. Overall, seven cases of nosocomial VZV infection (five patients and two nurses) and no deaths were identified in the present outbreak. No new cases of VZV were identified among the staff and patients of PMR ward during the next 42 days (twice the maximum incubation period) and outbreak was declared as controlled. The epidemiological curve and evolution of VZV outbreak in PMR ward are depicted in Figures 1 and 2, respectively.

## Discussion

VZV, belonging to family *Herpesviridae*, are enveloped virus with double-stranded DNA (dsDNA). The transmission is via aerosol and direct contact. Virus from an infected individual is shed from 1 to 2 days before rash onset until all the chickenpox lesions have crusted. VZV infection presents either as chickenpox or zoster. Chickenpox is classically described as a self-limiting childhood disease. Adults and immunocompromised individuals tend to have more severe disease often needing hospitalization. Zoster or shingles occur in adults following the reactivation of latent virus in the trigeminal nerve.

Nosocomial outbreaks involving patients and HCWs are being increasingly reported from diverse settings in India [4, 7–9]. Sharma et al. reported an outbreak of VZV among ICU staff of a tertiary care hospital in North India where three nurses and two resident doctors were infected [4]. A report from Rajasthan in western India reported an outbreak in transplant unit which affected 14 HCWs [8]. Eight cases of healthcare-associated chicken pox were identified within a week at an armed forces hospital in Delhi following exposure to an adult patient with extensive VZV disease [7]. Interestingly, cadaver-acquired infection in four medical students after attending an autopsy was reported by Paul et al. [9]. Outbreaks are reported more commonly from ICUs, oncology or transplant units where patients are often immunocompromised. This current study reports an outbreak identified in the physical medicine and rehabilitation ward where most patients are admitted for long-term care, however, they are not immunocompromised. Further, the outbreak evolved to spread from infected patients to susceptible HCWs.

The primary case was a housekeeping staff who had joined back work before the advised period of isolation after apparent clinical recovery. The infection subsequently spread to the patients admitted in the ward. VZV is transmitted to susceptible individuals, most commonly through direct contact with skin lesions [10]. Studies have also shown that transmission can occur when the aerosolized virus enters via the respiratory tract or mucosal surface like conjunctiva [11]. Airborne route has previously been reported as a common mode of transmission in nosocomial as well as localized community outbreaks [12, 13]. Both primary varicella infection and herpes zoster (shingles) can act as the source of infection [10]. In our study as well, infection was likely transmitted from primary care to patients via aerosols as the affected patients did not come in direct contact with the housekeeping staff. Subsequent transmission from patients to HCWs may have been either due to direct contact during patient care or via aerosols.

Infection control and prevention measures were undertaken promptly after the suspected outbreak was informed to the hospital infection control team. All probable cases were isolated in a cohort. The staff was re-educated about contact and airborne precautions including the use of N-95 masks during patient care. Samples were collected to identify the susceptible HCWs and vaccination initiated. Susceptible HCWs were reassigned to care for non-exposed patients and only immune HCWs were providing direct care for the infected patients [14]. These measures were partially successful in limiting the outbreak. Two out of three HCWs susceptible to VZV, subsequently became symptomatic after the first dose of vaccination. However, no further cases were identified amongst the admitted patients or other HCWs in the facility. Previous reports from India and other countries have similarly reported limited success in preventing the spread of occupational chickenpox outbreaks [1, 6–8, 15]. VZV is infectious in the incubation period and patients report non-specific symptoms early in the prodromal stage. This poses a challenge for infection control practitioners to identify cases early enough in the course of illness to prevent transmission to susceptible individuals in the close environment of wards and ICUs. Utpat et al. recently reported a nosocomial outbreak of VZV where nonimmune HCW contracted infection despite early patient isolation [6]. The problem is further exacerbated due to delay in procurement of vaccine in developing countries like India.

India has a high burden to childhood chickenpox. The age-related seroprevalence rate of anti VZV antibodies is reported as 29% in the age group of 1–5 years, 51.1% in 5–10 years, 71.7% in 11–15 years, 79.8% in 16–20 years, 88.1% in 21–30 years, and 91.1% in 31–40 years [16]. Another report by Inbaraj et al. found that 68.22%–76.9% of adult population from 18 to 39 (25.3 ± 4.3) years of age was immune to chickenpox [17]. However, lower seropositivities of 25.8% and 28% were reported in recent studies among university students of health sciences (mean age,21.6 years) and nurses (mean age, 21–30 years), respectively [18, 19]. WHO position paper has reported that VZV seropositivity as an evidence for immunity among HCWs varies region to region, from <5% in USA, 19% in Saudi Arabia, 26% in India to as high as 50% in Sri Lanka [20]. As only a quarter of HCWs in India are immune to VZV, healthcare facilities remain vulnerable to frequent outbreaks. Further, self-reported history of past infection with chickenpox had positive predictive value of only 82.4% [14]. In the present study as well, we found 10% (*n* = 3) of HCWs had no evidence of immunity to VZV and two of these had self-reported a past infection of chickenpox.

Prior to this outbreak, our institution did not have a policy defining the duration of leave for self-isolation in cases of transmissible infections among healthcare workers. The primary case was thus allowed to return to work before his lesions were fully scabbed. The outbreak resulted in a review of the institutional leave policy for HCWs infected with VZV as well as non-immune staff exposed to VZV case during the incubation period, to avoid further outbreaks. Prophylactic acyclovir has been recommended by some experts to prevent infections in immunosuppressed and pregnant contacts of VZV, where vaccine is contraindicated and can be considered in these groups [21].

## Limitations

Despite timely efforts to implement IPC measures to control the spread of nosocomial infection, there were certain limitations. During this current outbreak, we did not screen the patients for their antibody titres for their immune status as the test was not readily available at our hospital.

## Conclusion

This is the first report of VZV outbreak in a rehabilitation ward in India where patients admitted for long-term care and non-immune healthcare workers were infected. It highlights challenges to infection prevention in developing countries with limited resources like lack of negative pressure isolation room, non-availability of VZV immunoglobulin, and so on. These outbreaks also place an additional burden and expense on the healthcare system to investigate and control. In our report, the primary case (housekeeping staff) came back to work during the infectious period due to a lack of institutional leave policy for chickenpox cases. This highlights a need for a policy for the evaluation of immune status at the time of joining and vaccination of susceptible individuals and defined leave policy for infected staff. The information regarding immune status of the staff should be readily available electronically, so that it can easily accessed in the event of an outbreak. This can reduce response time during an outbreak where immune healthcare workers can be assigned for care of patients with varicella infection and non-immune exposed staff can be quarantined in a timely manner.

## Data Availability

Data sharing is not applicable to this article as no new data were created or analyzed in this study.
